# High-Pressure Dielectric Studies—a Way to Experimentally Determine the Solubility of a Drug in the Polymer Matrix at Low Temperatures

**DOI:** 10.1021/acs.molpharmaceut.1c00264

**Published:** 2021-07-11

**Authors:** Krzysztof Chmiel, Justyna Knapik-Kowalczuk, Ewa Kamińska, Lidia Tajber, Marian Paluch

**Affiliations:** † Department of Pharmacognosy and Phytochemistry, Faculty of Pharmaceutical Sciences in Sosnowiec, Medical University of Silesia in Katowice, ul. Jagiellońska 4, 41-200 Sosnowiec, Poland; ‡ Institute of Physics, Faculty of Science and Technology, University of Silesia, SMCEBI, 75 Pułku Piechoty 1a, 41-500 Chorzów, Poland; § School of Pharmacy and Pharmaceutical Sciences, Trinity College Dublin, 2 Dublin, Ireland

**Keywords:** ASD, dielectric spectroscopy, solubility, high-pressure, nimesulide, Kollidon VA64, Flory−Huggins theory

## Abstract

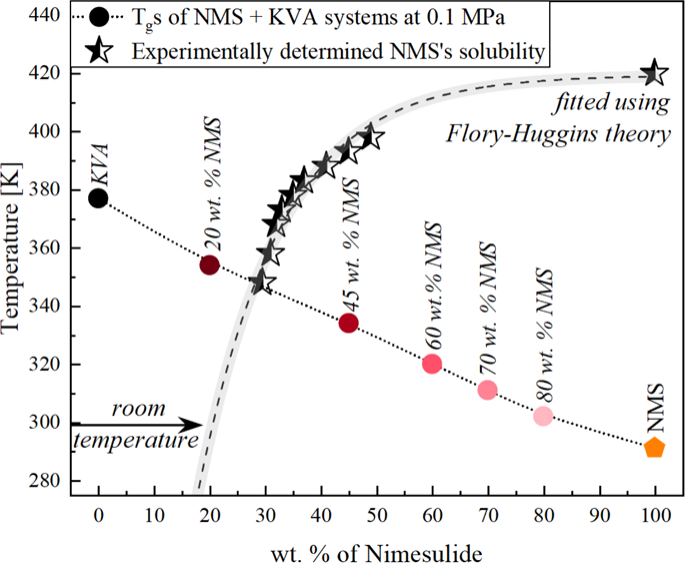

In this work, we employed broad-band dielectric spectroscopy to determine the solubility limits of nimesulide in the Kollidon VA64 matrix at ambient and elevated pressure conditions. Our studies confirmed that the solubility of the drug in the polymer matrix decreases with increasing pressure, and molecular dynamics controls the process of recrystallization of the excess of amorphous nimesulide from the supersaturated drug–polymer solution. More precisely, recrystallization initiated at a certain structural relaxation time of the sample stops when a molecular mobility different from the initial one is reached, regardless of the temperature and pressure conditions. Finally, based on the presented results, one can conclude that by transposing vertically the results obtained at elevated pressures, one can obtain the solubility limit values corresponding to low temperatures. This approach was validated by the comparison of the experimentally determined points with the theoretically obtained values based on the Flory–Huggins theory.

## Introduction

Developing a drug as an amorphous solid dispersion (ASD) has many benefits as over 80% of ASDs offers better bioavailability compared to the crystalline form of the active substance.^1^ However, one should keep in mind that this is not an easy task. The main challenge lies in identifying the optimal ratio between the active pharmaceutical ingredient (API) and the polymer, which provides the best dissolution, while maintaining the physical stability of the ASD. It should be highlighted that one can ensure the physical stability of the API–polymer mixture from a thermodynamic point of view. To do so, two main requirements must be met. The most important factor is to appropriately select the ingredients in a way to ensure that the drug can be dissolved in a polymer matrix as their mutual solubility/miscibility plays a key role. Next, it is to use the amount of the API, which does not exceed the solubility limit in a given polymer; thus, the API concentration must be maintained below the saturation level.^2−6^ Thus, the best approach to developing stable amorphous drug–polymer systems seems to be the determination of the solubility limit of the drug in a given polymer in advance. This will allow to identify the maximum amount of API that can be used without compromising the amorphous nature of the drug during the shelf-life.

A number of experimental approaches exploring the subject of drug–polymer solubility have been proposed.^2,7−13^ However, these methods are burdened with certain limitations. Solubility in small-molecule solutions is defined as an equilibrium thermodynamic parameter, that is, the chemical potential of the solute in the solid phase is the same as that in the liquid phase. One can extend this definition for polymer solvents supposing that equilibrium can be reached when the measurement is performed well above the *T*_g_ of the system. Thus, when the temperature is equal or lower than the glass-transition temperature (*T*_g_), the API–polymer system becomes too viscous to maintain the equilibrium. Therefore, the experimentally determined solubility in the context of amorphous substances is defined as the “apparent” solubility.^9,12,13^ However, it should be pointed out that there is a way to predict, at a broad temperature range (even to the extent not available experimentally), the solubility of an API in the polymer matrix. The most commonly used theoretical approaches are based on either (i) perturbed-chain statistical associating fluid theory^14−16^ or (ii) the Flory–Huggins (FH) theory.^17−22^

Hot-melt extrusion (HME) is a processing technique suitable for the production of ASDs for pharmaceutical applications.^23−25^ During the extrusion, a mix of the crystalline drug and the chosen polymer (raw materials) is subjected to shearing and intense mixing of a rotating screw under an elevated temperature and pushed through a die to obtain a product of homogeneous shape.^26,27^ The pressures generated within an extruder can reach very high values (up to 70 MPa).^28^ There are a number of reports addressing the compression-induced recrystallization of amorphous APIs, at conditions corresponding to the pressure exerted during extrusion.^29−31^ A good example of the discussed phenomenon is the study conducted on amorphous probucol. The authors confirmed that the amorphous drug does not exhibit any tendency toward devitrification up to the point where the elevated pressure was applied. A compression force equal to 10 MPa immediately induced devitrification of the amorphous API. Interestingly, once recrystallization started, the process progressed even when the initial crystallization-inducing factor was no longer present (when the sample was decompressed).^32^ Due to the fact that probucol was classified as a pressure-controlled compound,^33^ other drugs in this group might, but not necessarily, have to display a similar behavior. Another example of the drug with a well-documented sensitivity to elevated pressure is bicalutamide.^34,35^ Szafraniec-Szczęsny et al. have shown that the tendency of amorphous bicalutamide toward recrystallization increases with the increasing compression force,^36^ thus confirming the importance of the pressure factor when considering the stability of amorphous systems.

The latest studies have revealed information about the effect of the increased pressure on the earlier discussed solubility limit.^37,38^ However, our knowledge of this phenomenon is still limited. It should be noted that a simple comparison of the number of reports addressing the subject of drug–polymer solubility under ambient pressure conditions with those considering the effect of increased pressure reveals a substantial disproportion. This is surprising considering extrusion-based drug formulations.

In this paper, we determined the solubility limits of nimesulide (NMS) in the Kollidon VA64 (KVA) matrix at both ambient and elevated pressure conditions, starting from the supersaturated solutions. Most of the experimental approaches employed to determine the solubility limit are based on calorimetric measurements.^2,7−13^ Nevertheless, the results presented herein were obtained with the use of both differential scanning calorimetry (DSC) and broad-band dielectric spectroscopy (BDS). BDS is an experimental technique already applied to determine the solubility limit at ambient pressure.^39−43^ Moreover, due to the fact that this method has been repeatedly used to test amorphous materials under elevated pressures,^32,44^ in particular, as it has been recently shown to determine the aforementioned solubility under elevated pressure conditions,^37,38^ we chose it as the main experimental technique.

The aim of our investigations was to find the answer to the following question: How important are the solubility limit studies performed at elevated pressures and can we translate them to ambient pressure conditions? To achieve this goal, we conducted a series of DSC and BDS measurements. The thermal properties of ASDs containing various concentrations of NMS and KVA were investigated by DSC. The molecular mobility of the prepared drug–polymer mixtures, which was later used to determine the solubility limits at both ambient and elevated pressures, was measured by means of BDS. Furthermore, isothermal and nonisothermal measurements allowed us to determine the concentration dependencies of *T*_g_ of NMS + KVA systems.

## Experimental Section

### Materials

NMS with molecular weight *M*_W_ = 308.31 g mol^–1^ and purity ≥ 99% was supplied by Kemprotec Limited (UK). Polyvinylpyrrolidone vinylacetate–Kollidon VA64 (KVA) of molecular mass *M*_W_ = 45,000–47,000 g mol^–1^ was purchased from BASF SE (Ludwigshafen, Germany). The NMS and KVA samples were received as a light-yellow and white powder, respectively. Both NMS and KVA were used without further purification.

### Preparation of Binary Systems

To acquire homogeneous samples, the API and the polymer were mixed at appropriate ratios in a mortar. The sample preparation for ambient and high-pressure BDS measurements involved melting at *T* = 433 K followed by vitrification on a previously chilled copper plate. All measurements were performed immediately after the preparation of the amorphous systems to avoid recrystallization.

### Differential Scanning Calorimetry

The thermal properties of NMS-based ASDs were examined using a Mettler-Toledo DSC 1 STARe System (Columbus, OH, USA). The measuring device was equipped with a HSS8 ceramic sensor with 120 thermocouples. The instrument was calibrated for temperature and enthalpy using indium and zinc standards. Crystallization and melting points were determined as the onset of the peak, whereas the glass-transition temperature as the midpoint of the heat capacity increment. The samples were measured in a closed aluminum crucible (40 μL) with a pinhole to prevent the buildup of pressure. Amorphous samples were obtained *in situ* in the DSC apparatus by fast cooling (at the rate of 20 K/min) of the previously melted sample (data not shown). All measurements were carried out in the range from 270 to 433 K using a 10 K/min heating rate and a 20 K/min cooling rate under a nitrogen purge (60 mL/min).

### Broad-band Dielectric Spectroscopy

The dielectric measurements of NMS-based ASDs were carried out using a Novo-Control GMBH Alpha dielectric spectrometer (Montabaur, Germany) in the frequency range from 10^–1^ to 10^6^ Hz at preselected temperatures at a heating rate of 2 K/min (and a cooling rate of 50 K/min in case of quenching samples on a copper plate after melting and 10 K/min after annealing at certain temperatures). The temperature was controlled by a Quatro temperature controller with temperature stability better than 0.1 K. Dielectric studies of NMS and NMS + KVA binary systems were performed immediately after their vitrification by the fast cooling of the melt in a parallel-plate cell made of stainless steel (15 mm diameter and 0.1 mm gap with a quartz spacer).

For high-pressure BDS measurements, a high-pressure Unipress U111 setup (Warszawa, Poland) was additionally employed. For these experiments, the sample was measured in a similar parallel-plate cell made of stainless steel (15 mm diameter and 0.1 mm gap with a Teflon spacer). The cell was then sealed and encased in a Teflon tape to separate it from the silicon liquid. The temperature was adjusted with a precision of 0.1 K by a Julabo heating circulator (Seelbach, Germany).

The protocol used to determine solubility was as follows:^45^

(i) In the first step, a strongly supersaturated glass solution is formed. First, a physical mix is obtained by mixing the API and polymer at appropriate ratios in a mortar for 20 min to acquire a homogeneous sample. Then, for both ambient and high-pressure measurements, the sample is melted and vitrified using well-defined heating and cooling rates.
(ii) The supersaturated glass solution is then loaded into a DSC/BDS instrument and annealed above its glass-transition temperature to reach the equilibrium concentration at the annealing temperature. Depending on the sample and measuring technique, a sufficient annealing time must be determined to reach this equilibrium concentration in a wide range of annealing temperatures. In this work, this annealing time was up to 50 and 70 h for DSC and BDS measurements, respectively. This difference was mainly due to the geometry of the container used in both experiments, as it is one of the factors affecting the beginning and progress of the crystallization process.^46^
(iii) In the third step, the equilibrium concentration of the drug remaining in the polymer matrix after annealing is determined. This is done by determining the glass-transition temperature of the annealed material by DSC/BDS.
(iv) The last step is determination of the sample composition by comparing its glass transition to the experimentally determined compositional dependence of *T*_g_’s (determined for each measuring technique independently).

By repeating steps (ii–iv) for different annealing temperatures, it is possible to determine the solubility curve.

### True Density Measurements

The true density of amorphous NMS was measured by an AccuPyc 1330 pycnometer, Micromeritics, using helium (99.995% purity) to determine the volume of the sample.^19^ A 1 cm^3^ aluminum sample cup was used. The instrument was calibrated immediately before the analysis, and the measurement comprising five volume determinations was performed in duplicate.

### Mathematical Modeling

Modeling of the phase diagrams and statistical analysis were performed in Origin 2018. Nonlinear least-squares curve fits to experimentally determined data were obtained by applying the Levenberg–Marquardt iteration algorithm until the Chi-square tolerance value of 1 × 10^–9^ was reached and the fit converged.^19^ No weighting for the parameters was applied.

## Results and Discussion

### Determination of the Solubility Limits of the NMS + KVA ASD via Calorimetric Measurements

**Figure 1:**
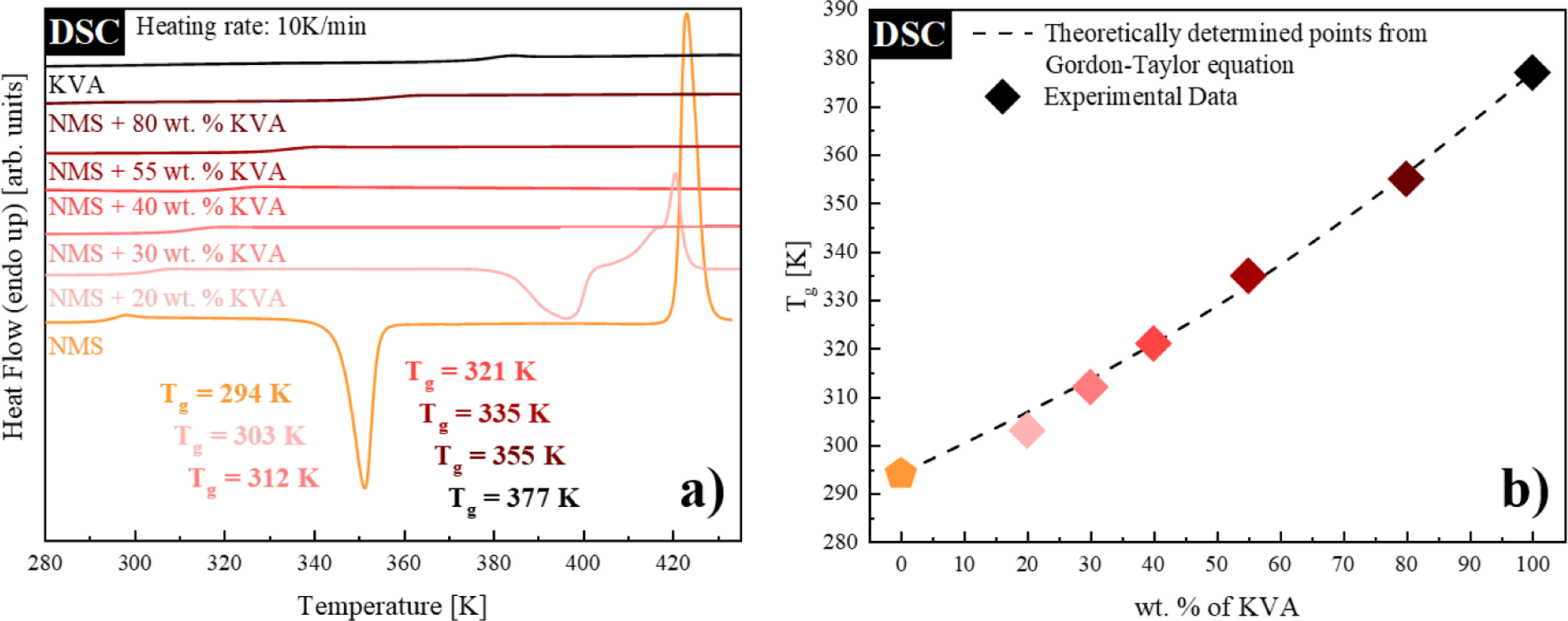
(a) Thermograms of amorphous: NMS^47^ (orange), NMS + 20 wt % KVA (pink), NMS + 30 wt % KVA (raspberry), NMS + 40 wt % KVA (red), NMS + 55 wt % KVA (cardinal), NMS + 80 wt % KVA (burgundy), and KVA (black). (b) Glass-transition temperatures of NMS + KVA mixtures. Diamonds correspond to the experimentally determined *T*_g_ values, while the orange pentagon (NMS) represents the experimental data corresponding to the literature. The dashed line represents the Gordon–Taylor prediction.

The thermal properties of neat NMS have been already documented in the literature;^47^ therefore, we focused mainly on its binary systems. The thermograms of the amorphous NMS + KVA mixtures at various weight concentrations (after melting and quenching in the DSC apparatus), obtained through measurements carried out in the range from 270 to 433 K at the 10 K/min heating rate, are presented in panel (a) of Figure 1. All measured systems are characterized by a single-step thermal event that corresponds to the glass transition. Considering the work of Baird and Taylor, the presence of two separate *T*_g_’s during the standard DSC measurement would correspond to the phase separation in the sample.^48^ Therefore, the obtained results imply the homogeneity of the systems.^49,50^ The exact values of *T*_g_ are as follows: 294 K (NMS),^47^ 303 K (NMS + 20 wt % KVA), 312 K (NMS + 30 wt % KVA), 321 K (NMS + 40 wt % KVA), 335 K (NMS + 55 wt % KVA), 355 K (NMS + 80 wt % KVA), and 377 K (KVA).

One can observe a continuous evolution of *T*_g_ with the increasing amount of the polymer in the system. This phenomenon is common, and its origin can be explained by either the antiplasticization effect exerted by the addition of the polymer with a higher *T*_g_ value than that of a neat amorphous drug or the specific interactions between the components.^51^ If the antiplasticization effect is dominant, *T*_g_ of the mixtures should vary with the polymer content in accordance to the Gordon–Taylor (G–T) prediction, which is defined as follows
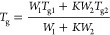
1where *T*_g_, *T*_g1_, and *T*_g2_ are the glass-transition temperatures of the drug–polymer mixture, the amorphous drug, and the polymer, respectively; *W*_1_ and *W*_2_ are the weight fractions of the drug and the polymer, respectively; and *K* is a parameter that can be calculated according to the formula
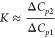
2where Δ*C*_*p*1_ and Δ*C*_*p*2_ are the changes in heat capacity at *T*_g_ of the drug and polymer, respectively. In panel (b) of Figure 1, *T*_g_ values predicted from the G–T equation (eq 1) (marked as black dashed line) were compared with the experimentally obtained *T*_g_’s of the NMS + KVA systems containing various polymer concentrations (marked as diamonds). It can be seen that they are in good agreement. Such a result indicates that the drug should be miscible and homogeneously dispersed in the polymer.^52^ This is of importance as highly miscible systems are usually characterized by better physical stability. It should be noted that although it is not a conclusive proof, no significant deviation of the experimental points from the G–T prediction suggests the lack of any specific strong drug–polymer interactions.^52^

**Figure 2:**
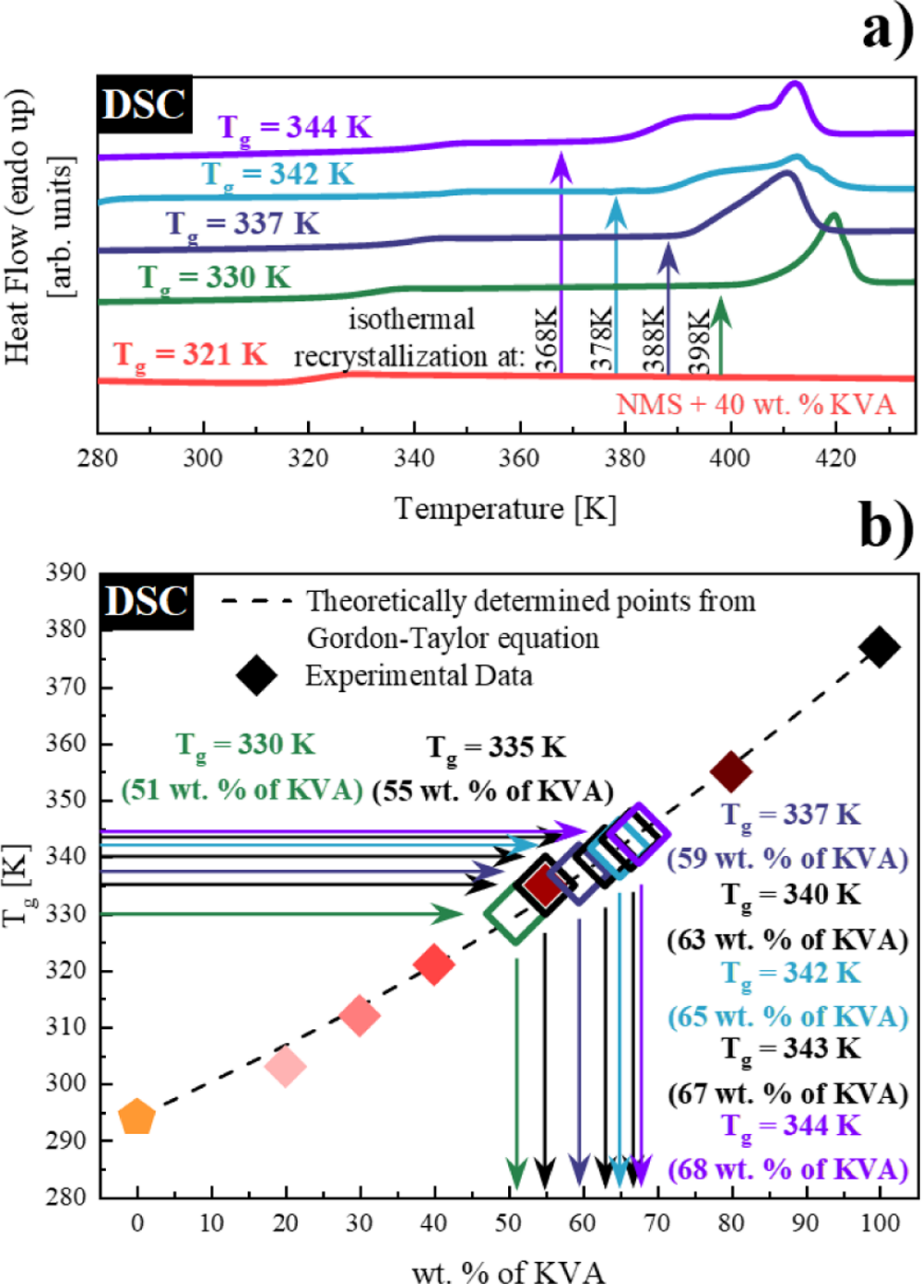
(a) Thermograms of the fully amorphous NMS + 40 wt % KVA mixture (red) as well as the sample after isothermal recrystallization at: 398 K (green), 388 K (dark blue), 378 K (turquoise), and 368 K (violet). (b) Glass-transition temperatures of NMS + KVA mixtures. Diamonds correspond to the experimentally determined *T*_g_ values, while the orange pentagon (NMS) represents the data taken from the literature. The dashed line represents the Gordon–Taylor prediction. Empty diamonds correspond to the *T*_g_ values obtained after recrystallization performed at (from the right side): 368 K (violet), 373 K (black), 378 K (turquoise), 383 K (black), 388 K (dark blue), 393 K (black), and 398 K (green).

The determination of the solubility limit presented in this work, regardless of the applied experimental technique (whether calorimetric or dielectric), is based on Mahieu’s approach.^2^ The amorphous NMS + 40 wt % KVA system was arbitrarily chosen for the subsequent solubility limit studies as the supersaturated starting composition. According to the protocol proposed in ref (2), the samples were annealed at the following temperatures: 368, 373, 378, 383, 388, 393, and 398 K for up to 50 h and then cooled down and re-measured by DSC to determine *T*_g_. Panel (a) of Figure 2 shows representative examples of the samples re-measured after isothermal recrystallization at four out of seven temperatures selected above.

Determination of the exact concentration of the remaining amorphous systems after recrystallization was possible based on the obtained *T*_g_ values. This can be done by the simple comparison of these values to either the experimentally determined dependency of *T*_g_ on the concentration or to the G–T prediction, as no discrepancies between them were noticed [see panel (b) of Figure 2]. Both the *T*_g_’s as well as solubility limit values determined after the recrystallization of the excess amount of drug from the NMS + 40 wt % KVA mixture are collected in Table 1. The obtained results served as a verification of the data obtained from the dielectric studies.

**Table 1:** Calorimetric Glass-Transition Temperatures Determined after the Recrystallization of the Excess Amount of the Drug from the NMS + 40 wt % KVA System at Various Temperatures and the Corresponding Drug Concentrations of the NMS + KVA ASDs

temperature of performed recrystallization [K]	368	373	378	383	388	393	398
*T*_g_ [K]	344 ± 0.5	343 ± 0.5	342 ± 0.5	340 ± 0.5	337 ± 0.5	335 ± 0.5	330 ± 0.5
solubility limit (wt % of API in the system)	32 ± 0.2	33 ± 0.2	35 ± 0.2	37 ± 0.2	41 ± 0.2	45 ± 0.3	49 ± 0.2

### Determination of the Solubility Limits of the NMS + KVA ASD via Dielectric Measurements at Ambient Pressure Conditions

**Figure 3:**
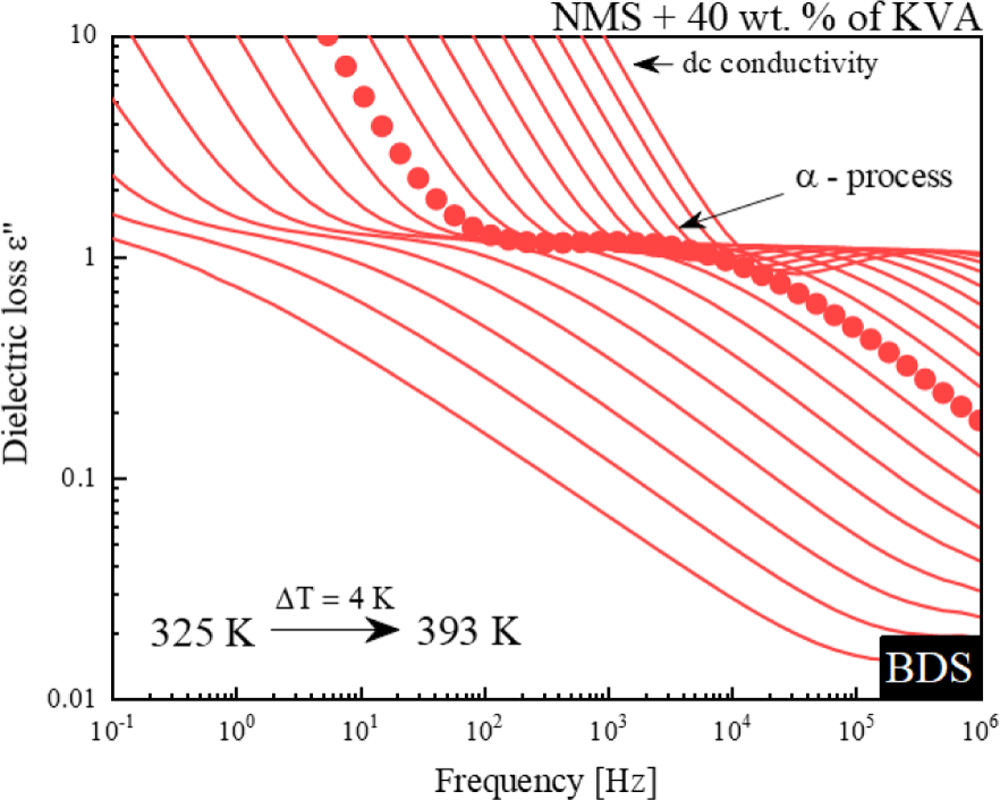
Representative dielectric loss spectra of the amorphous binary mixture of NMS + 40 wt % KVA above its glass-transition temperature (from 325 to 393 K, recorded every 4 K), collected at ambient pressure (0.1 MPa). Red lines indicate the α process.

Similar to the solubility limits determined by DSC, the experimental determination of the concentration dependency of the glass-transition temperature was first determined by BDS. This dependence was then used to identify the compositions obtained after recrystallization. For this purpose, the analysis of the molecular mobility of each prepared sample was undertaken as a series of BDS measurements. The representative dielectric loss spectra of the NMS + 40 wt % KVA mixture, which were obtained during the heating of the amorphous sample, above its *T*_g_, are shown in Figure 3. The spectra showed one loss peak corresponding to the structural α relaxation. The maximum of this peak moves toward higher frequencies with the increasing temperature. The intensity of this peak [its dielectric strength (Δε)] is proportional to the number of units involved in the structural relaxation. Therefore, a sudden drop of Δε would reflect the onset of the sample recrystallization.^53,54^ Due to the fact that no sudden drop in intensity was observed on the dielectric loss spectra up to 393 K, one can assume that the recrystallization process did not occur during nonisothermal experiments (see Figure 3). This can indicate the relatively high physical stability of the NMS-based ASD containing not less than 40 wt % of KVA.

**Figure 4:**
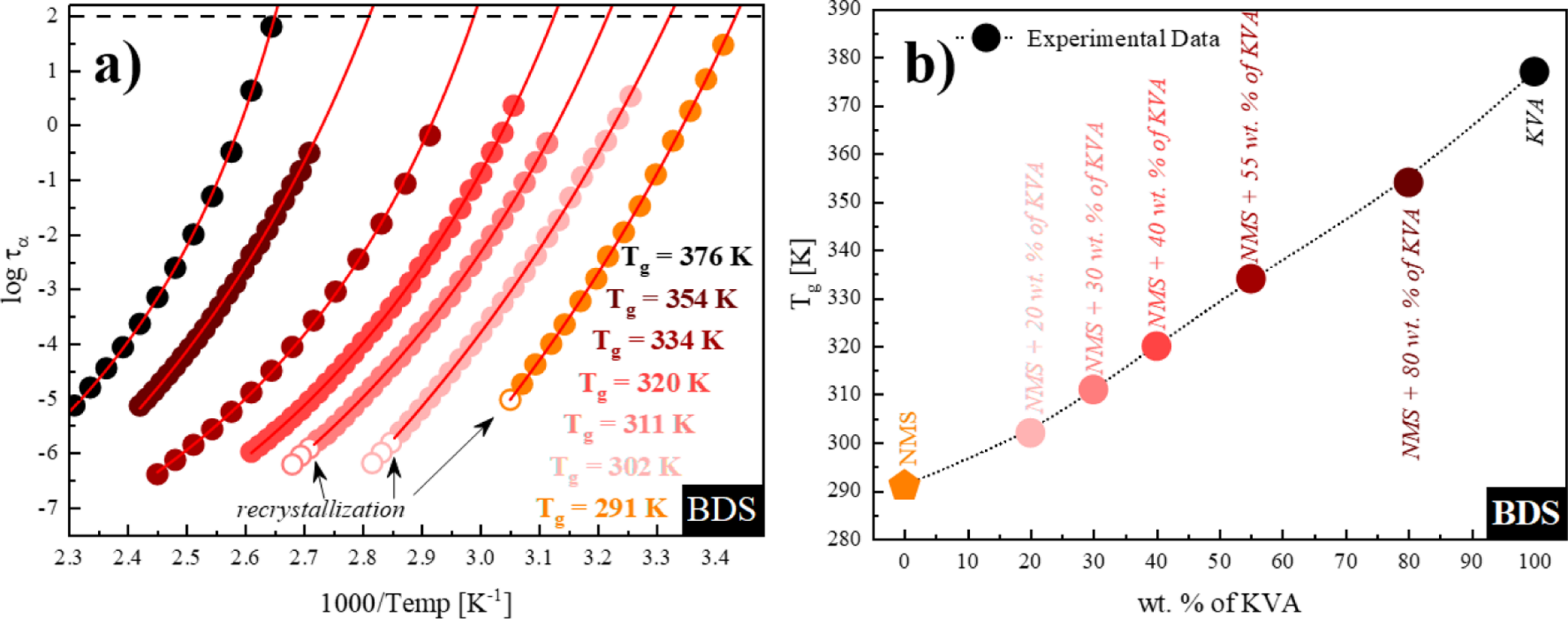
(a) Relaxation map of the following systems (from the right side): NMS (orange), NMS + 20 wt % KVA (pink), NMS + 30 wt % KVA (raspberry), NMS + 40 wt % KVA (red), NMS + 55 wt % KVA (cardinal), NMS + 80 wt % KVA (burgundy), and KVA (black). Empty circles indicate the crystallization onset. Temperature dependences of τ_α_ in the supercooled liquid are described by the VFT equation (red solid lines). (b) Glass-transition temperatures of NMS + KVA mixtures. The circles correspond to the experimentally determined *T*_g_ values, while the orange pentagon (NMS) represents the data taken from the literature.^47^

From the analysis of the loss spectra collected above *T*_g_, we determined the temperature dependencies of the α-relaxation times for all the measured systems [see panel (a) of Figure 4]. To obtain the values of τ_α_ at various temperatures, the experimental data were fitted using the Havriliak–Negami (HN) function (eq 3)^55^
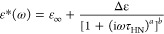
3where ε_∞_ is the high-frequency limit permittivity, ε_0_ is the permittivity of vacuum, Δε is the dielectric strength, ω is equal to 2π*f*, τ_HN_ is the HN relaxation time, and *a* and *b* represent the symmetric and asymmetric broadening of the relaxation peak, respectively. Based on the fitting parameters determined above, the values of τ_α_ were calculated by means of the following formula
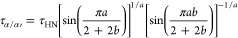
4

Relaxation times, obtained from the fitting procedure described above, are presented as circles in panel (a) of Figure 4. It can be seen that the τ_α_(*T*) values determined for the measured systems show a non-Arrhenius behavior. Therefore, we parameterized them using the Vogel–Fulcher–Tamman (VFT) equation^56−58^
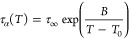
5where τ_∞_, *B*, and *T*_0_ are the fitting parameters. By extrapolating the VFT fit to 100 s, we were able to determine the *T*_g_ values [according to the definition *T*_g_ = *T*(τ_α_ = 100 s)] of the following systems: NMS, NMS + 20 wt % KVA, NMS + 30 wt % KVA, NMS + 40 wt % KVA, NMS + 55 wt % KVA, NMS + 80 wt % KVA, and neat KVA as 291, 302, 311, 320, 334, 354, and 376 K, respectively. The obtained results were in good agreement with the calorimetric data, even considering the different heating rates applied during both analyses: 10 K/min in the case of DSC and 2 K/min in the case of BDS. The theoretical explanation of this phenomenon as well as calculations considering the employed experimental techniques is well described in ref (59). Panel (b) of Figure 4 shows the experimentally determined compositional dependence of the *T*_g_ values. As stated at the beginning of this section, this was then used to identify the compositions obtained after the recrystallization of the excess amount of NMS from the supersaturated solutions.

**Figure 5:**
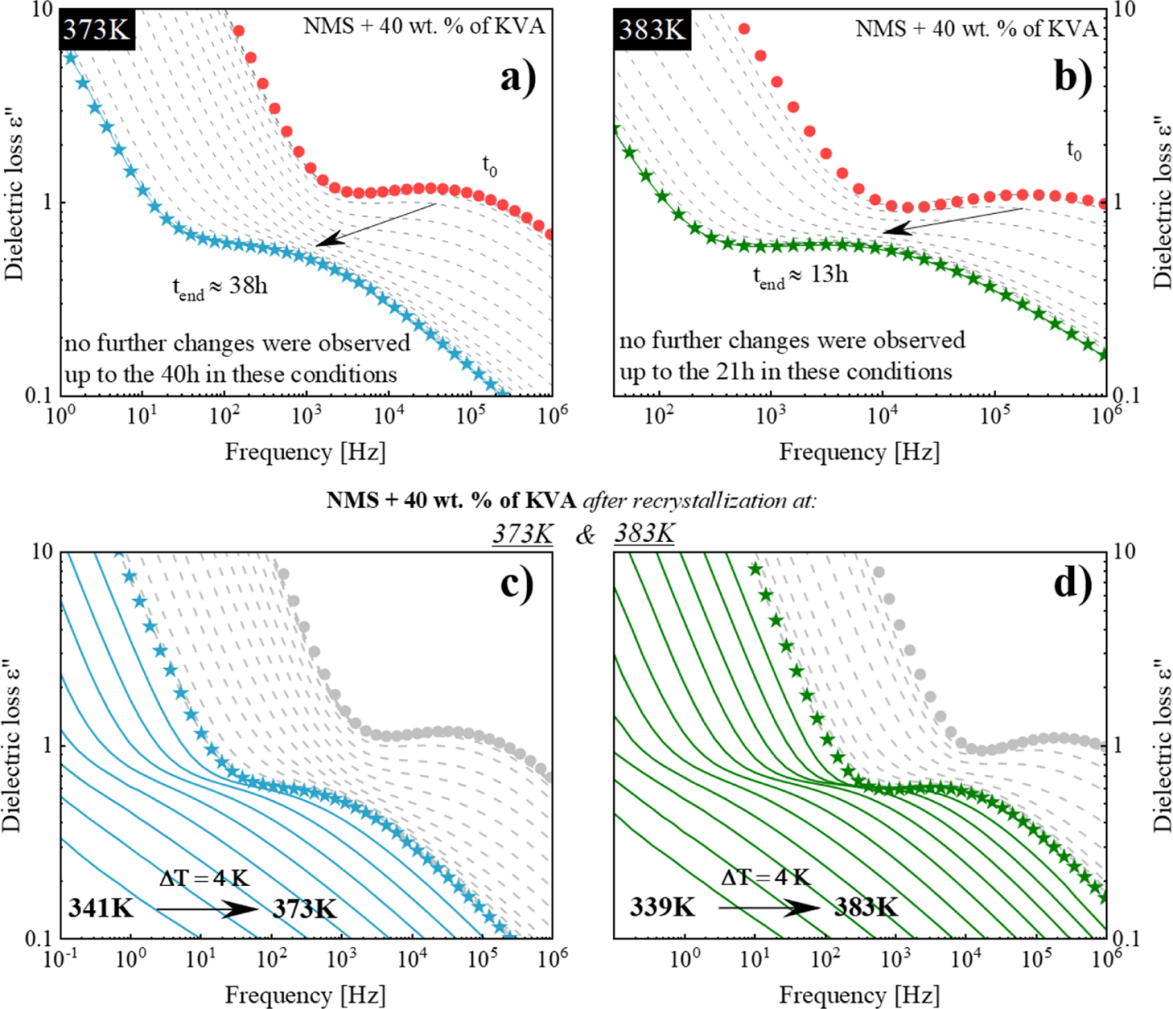
(a,b) Dielectric spectra obtained during the isothermal recrystallization determined at 373 and 383 K, respectively. *t*_0_ indicates the first recorded spectrum at the set temperature. (c,d) Dielectric spectra obtained during additional measurements in the temperature range from 341 to 373 and 339 to 383 K, registered every 4 K, carried out after isothermal recrystallization at 373 and 383 K, respectively.

Similar to the calorimetric measurements carried out in the first part of this work, the initial (supersaturated) drug–polymer composition containing 40 wt % of KVA was chosen for the subsequent solubility studies. In the first step of our investigations, the sample was annealed at seven different temperatures from 368 to 398 K (every 5 K) for up to 70 h. Panels (a,b) of Figure 5 show the dielectric spectra of the representative examples of NMS + 40 wt % KVA during the isothermal recrystallization performed at two temperatures: 373 and 383 K, respectively. As mentioned earlier, the recrystallization of the amorphous material, registered during the isothermal dielectric studies, is manifested as the drop in the intensity of the α-process peak over time. However, in the presented case of the recrystallization of the amorphous NMS from the drug–polymer ASD, one can additionally notice a shift of the structural relaxation process toward lower frequencies. This can be explained by the decrease in the global mobility of the system associated with an increase in the *T*_g_ value of the ASD due to the decreasing amount of the amorphous drug remaining in the mixture (the weakening of the plasticization effect). This phenomenon has been observed for systems comprising a low-*T*_g_ API and a high-*T*_g_ polymer. A similar behavior was also observed during the recrystallization of flutamide in the flutamide–Kollidon VA64 system,^37^ aripiprazole in compositions with Kollidon VA64 or Soluplus,^41^ and sildenafil in the sildenafil + Kollidon VA64 ASD system.^43^ It is worth mentioning that the opposite phenomenon, the shift of the α-relaxation process toward higher frequencies during the recrystallization corresponding to the enhancement of the plasticizing effect, can be observed in the systems based on a high-*T*_g_ API and a low-*T*_g_ polymer such as sildenafil + poly vinylacetate.^43^ When the recrystallization of the excess amount of NMS from the supersaturated solution had ceased, one loss peak could be observed in the dielectric loss spectrum (see panels (a,b) of Figure 5).

**Figure 6:**
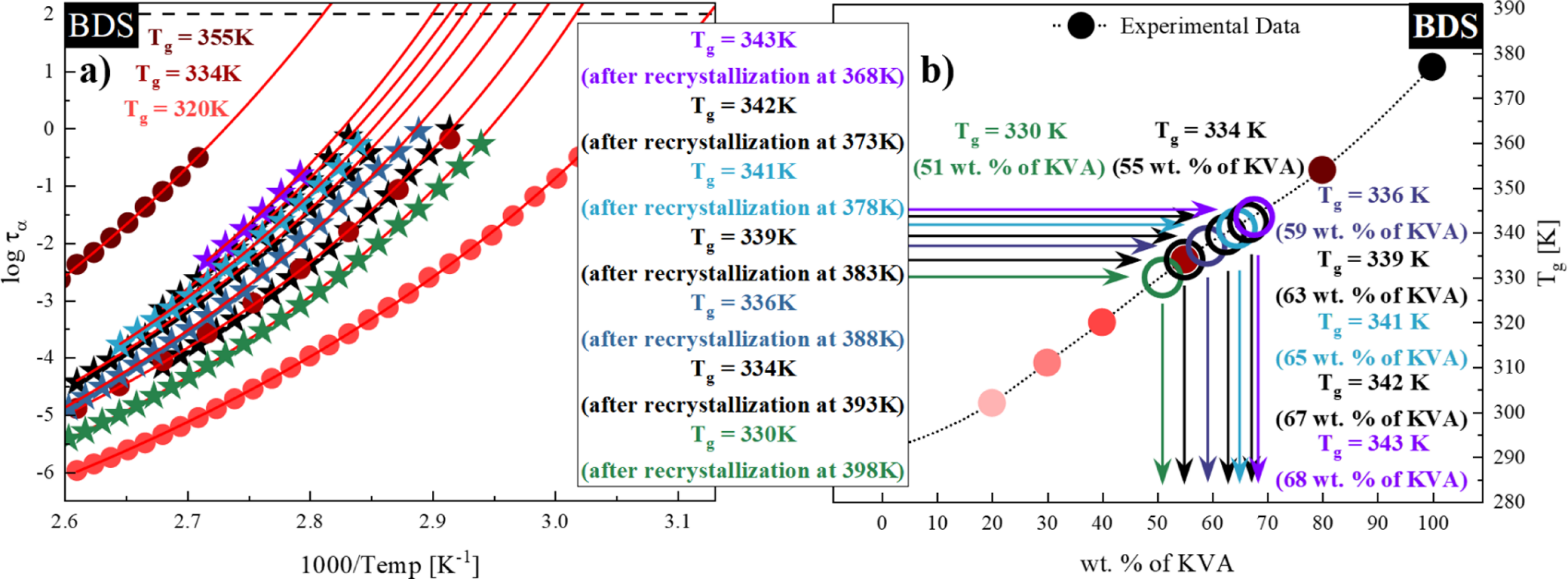
(a) Relaxation map of the fully amorphous system (from the right side): NMS + 40 wt % KVA (red circles), NMS + 55 wt % KVA (cardinal circles), NMS + 80 wt % KVA (burgundy circles), and system after recrystallization at (from the right side): 398 K (green stars), 393 K (black stars), 388 K (dark blue stars), 383 K (black stars), 378 K (turquoise stars), 373 K (black stars), and 368 K (violet stars). Temperature dependences of τ_α_ in the supercooled liquid were described by the VFT equation (red solid lines). (b) Glass-transition temperatures of NMS + KVA mixtures. Full circles correspond to the experimentally determined *T*_g_ values. Empty circles correspond to the *T*_g_ values obtained after recrystallization performed at (from the right side): 368 K (violet), 373 K (black), 378 K (turquoise), 383 K (black), 388 K (dark blue), 393 K (black), and 398 K (green).

Next, we performed additional experiments, in which the sample after recrystallization was remeasured during heating to determine the molecular dynamics of the system in a broad temperature range [see panels (c,d) of Figure 5]. Then, the obtained spectra were analyzed and subjected to the HN fitting procedure to determine the temperature dependence of the relaxation times of the NMS + KVA systems after recrystallization (τ_α′_(*T*); see panel (a) of Figure 6. To determine *T*_g_ of the sample obtained after isothermal recrystallization, we extrapolated the VFT fits to 100 s (*T*_g_ = *T*(τ_α_ = 100 s)). The *T*_g_ values determined after the recrystallization of the excess amount of the drug from the NMS + 40 wt % KVA mixture are listed in Table 2.

**Table 2:** Glass-Transition Temperatures Determined by BDS after the Recrystallization of the Excess Amount of the Drug from the NMS + 40 wt % KVA System at Various Temperatures and Corresponding Drug Concentrations of the NMS + KVA ASDs

temperature of performed recrystallization [K]	368	373	378	383	388	393	398
*T*_g_ [K]	343 ± 1.1	342 ± 1.0	341 ± 0.9	339 ± 0.8	336 ± 0.8	334 ± 0.7	330 ± 0.6
solubility limit (wt % of API in the system)	32 ± 0.9	33 ± 0.8	35 ± 0.8	37 ± 0.6	41 ± 0.5	45 ± 0.4	49 ± 0.4

As shown in panel (b) of Figure 6, we were able to identify the concentrations of the systems obtained after recrystallization by correlating the *T*_g_ value to the experimentally determined compositional dependence of the *T*_g_’s. The exact values are summarized in Table 2. It can be concluded that both sets of *T*_g_’s and the solubility limits obtained from the calorimetric and dielectric measurements are in excellent agreement.

As the experimental determination of the solubility limit has its limitations (i.e., high viscosity of the system at relatively low temperatures),^9,12,13^ to obtain another estimation of the solubility limit of NMS in the KVA matrix at room temperature, we decided to apply one of the theoretical approaches.

### Determination of the Solubility Limits of the NMS + KVA ASD at Ambient Pressure Conditions Using the FH Theory

Solubility of NMS in the KVA matrix at 298 K was determined using the F–H theory by measuring the parameter χ (the F–H interaction parameter) from the solid–liquid line using the following equation (eq 6)^60^
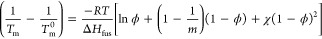
6where *T*_m_ and *T*m^0^ are the melting points of the API in the binary mixture and neat drug, respectively, *R* is the gas constant, *T* is the temperature, Δ*H*_fus_ is the heat of fusion of the pure drug, ϕ is the volume fraction of the API (NMS), *m* is the volume ratio of the polymer to drug, and χ is the F–H interaction parameter. Note that *m* can be calculated as per eq 7, where *M*_w_ is the molecular weight and *d* is the true density
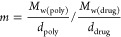
7

The following parameters were used in eqs 6 and 7: *T*_m_^0^ = 421 K, Δ*H*_fus_ = 32,988 J/mol, *d*_drug_ = 1.41 g/cm^3^, *d*_poly_ = 1.20 g/cm^3^, *M*_w(drug)_ = 308.3 g/mol, and *M*_w(poly)_ = 46,000 g/mol.

We employed two approaches in relation to the estimation of the χ parameter: (I) best fitting a single χ value to the experimental data points^61^ and (II) determining the temperature dependence of the interaction parameter χ^19^ (eq 8)
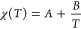
8where *A* and *B* are constants related to entropy and enthalpy contributions, respectively.^62^

**Figure 7:**
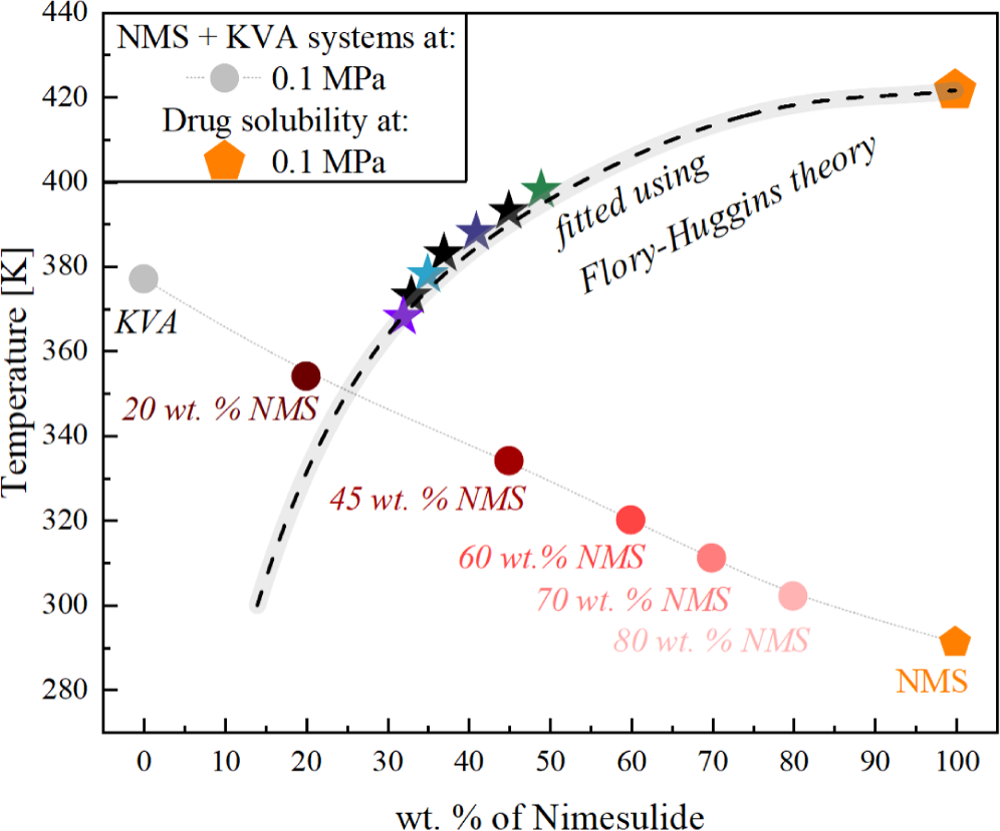
Glass-transition temperatures of NMS + KVA mixtures. Circles and stars correspond to the experimentally determined *T*_g_ and solubility limit values, respectively, while orange pentagons (NMS) represent experimental data that correspond to the literature.^47^ Dashed line represents drug solubility (the solid–liquid line) in the polymer obtained using theFH theory.

Using the approach (I), we determined the χ parameter as −1.17 ± 0.08, implying the miscibility of the drug and polymer. The solubility of NMS in KVA at 298 K was found to be 2.9 wt %. However, as can be seen in Figure 7, when determining the *A* and *B* parameters [approach (II)], estimated to be 6.695 ± 0.85 and −2996 ± 324, respectively, the solubility of NMS in the polymer was assessed to be 14 wt %.

Once we obtained the temperature dependence of the NMS solubility in the KVA matrix, we moved to high-pressure measurements to assess whether or not elevated pressure would affect it.

### Determination of the Solubility Limits of the NMS + KVA ASD via Dielectric Measurements at Elevated Pressure Conditions

**Figure 8:**
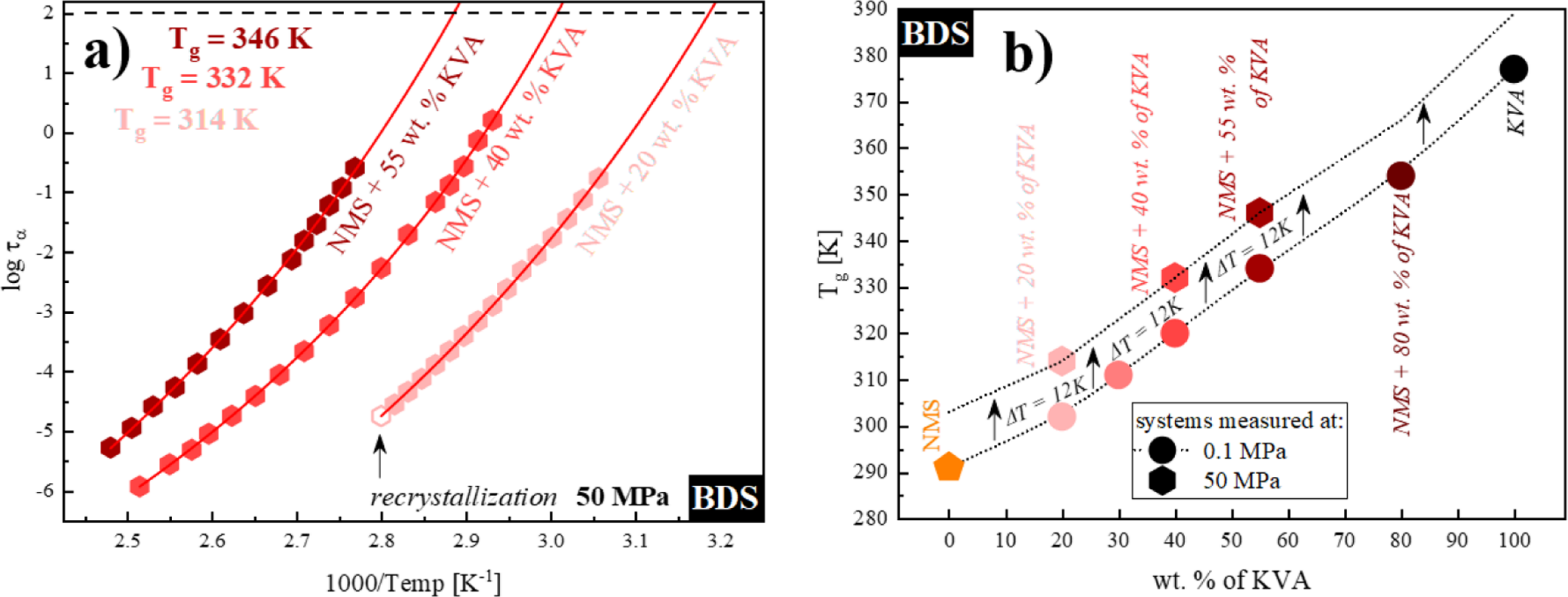
(a) Relaxation map of the following systems (from the right side): NMS + 20 wt % KVA (pink), NMS + 40 wt % KVA (red), and NMS + 55 wt % KVA (cardinal). Empty hexagon corresponds to the crystallization onset. Temperature dependences of τ_α_ in the supercooled liquid were described by the VFT equation (red solid lines). (b) Glass-transition temperatures of NMS + KVA mixtures. Hexagons and circles correspond to the experimentally determined *T*_g_ values at 50 and 0.1 MPa, respectively, while orange pentagons (NMS) represent the data taken from the literature. The dotted line is the vertically transposed relationship of the *T*_g_ dependence obtained at ambient pressure.

Similar to the measurements performed at atmospheric pressure presented in the previous section, we started with the determination of the molecular dynamics of the systems at elevated pressure conditions (at 50 MPa). The spectra exhibited one loss peak corresponding to the structural α relaxation (data not shown). From the analysis of these spectra collected above *T*_g_, we determined the temperature dependencies of the α-relaxation times for the measured systems. As can be seen in panel (a) of Figure 8, we estimated the *T*_g_ values of NMS + 20 wt % KVA, NMS + 40 wt % KVA, and NMS + 55 wt % KVA as 314, 332, and 346 K, respectively, from the extrapolation of VFT fit to 100 s. The nearly constant increase in *T*_g_ (Δ*T* = 12 K) is consistent with the high-pressure data obtained for another pharmaceutical, flutamide.^37,38^ This behavior can be explained by the decrease in the molecular mobility resulting from the increased densification of molecular packing of the system as the applied pressure raises.^63,64^ Observing the constant increase in *T*_g_, we decided to vertically displace the compositional dependence of the *T*_g_ values obtained at ambient pressure to this relationship. The result of this procedure can be seen in panel (b) of Figure 8. It should be mentioned that due to the equipment limitation of the used heating circulator, not all prepared samples could be measured, which resulted in a limited number of *T*_g_ values determined at elevated pressures.

**Figure 9:**
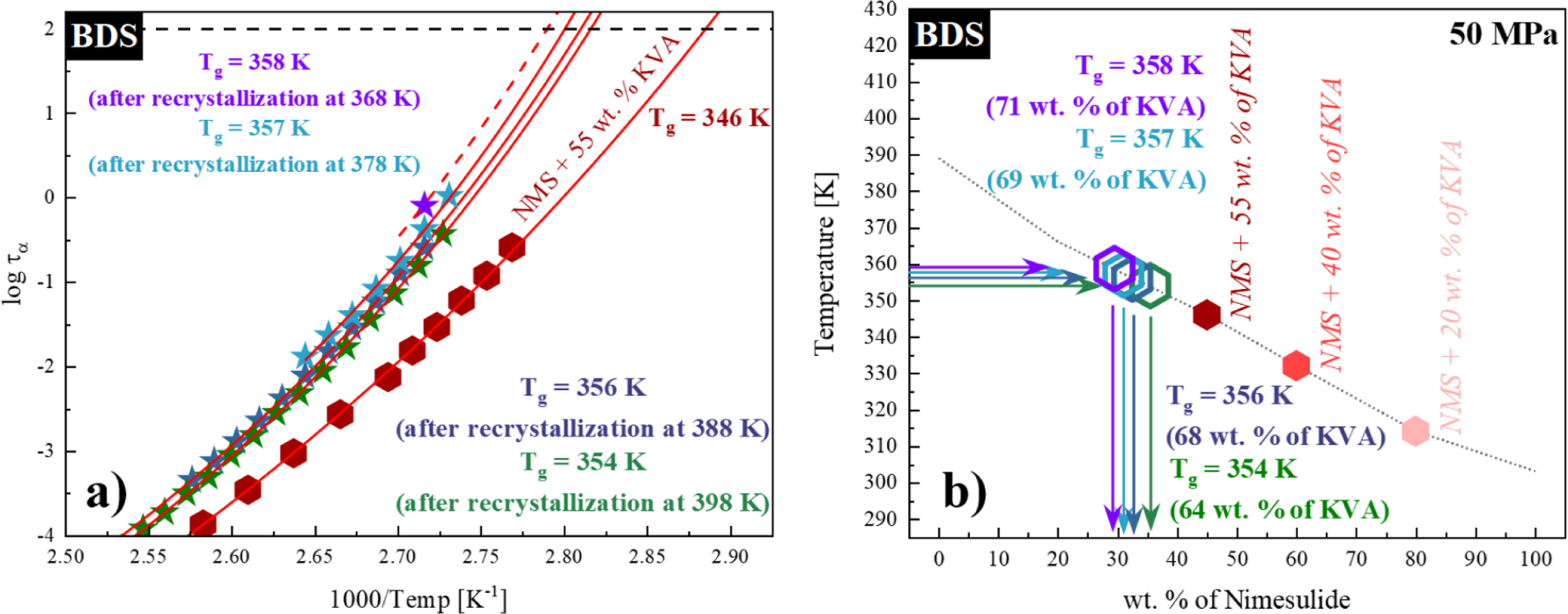
(a) Relaxation map of the fully amorphous NMS + 55 wt % KVA (cardinal hexagon) system and systems after recrystallization at (from the right side): 398 K (green stars), 388 K (dark blue stars), 378 K (turquoise stars), and 368 K (violet stars) at 50 MPa. The temperature dependence of τ_α_ in the supercooled liquid was described by the VFT equation (red solid lines). The red dashed line is the horizontal displacement of the VFT fit of the nearest parameterized concentration. (b) Glass-transition temperatures of NMS + KVA mixtures. Full hexagons correspond to the experimentally determined *T*_g_ values at 50 MPa. Empty hexagons correspond to the *T*_g_ values obtained after recrystallization performed at (from the left side): 368 K (violet), 378 K (turquoise), 388 K (dark blue), and 398 K (green). The dotted line is the vertically transposed relationship of the *T*_g_ dependence obtained at ambient pressure.

We next annealed the NMS + 40 wt % KVA system at 368, 378, 388, and 398 K for up to 100 h. When the recrystallization of the excess amount of NMS from the supersaturated solution had ceased, the samples were cooled down and then re-measured to determine the molecular dynamics of the systems in a broad temperature range (data not shown). By following the procedure already discussed in this work [extrapolation of the VFT fits to 100 s; *T*_g_ = *T*(τ_α_ = 100 s)], we were able to determine the corresponding *T*_g_’s [see panel (a) of Figure 9].

It has to be pointed out that in the case of the isothermal recrystallization performed at 368 K, only one dielectric spectrum could have been fitted with the acceptable accuracy by the HN function. Thus, the number of experimentally determined relaxation times was not sufficient to parameterize it with the VFT equation. However, due to the close proximity of the well-parameterized system, NMS + 40 wt % KVA, after annealing at 378 K, we shifted its VFT fit horizontally and used this extrapolation to determine *T*_g_ [red dashed line in panel (a) of Figure 9]. This approach has been validated as presented in our previous work on flutamide-based ASDs.^37,38^ The *T*_g_ values determined after the recrystallization of the excess amount of drug from the NMS + 40 wt % KVA mixture at an elevated pressure (50 MPa) are listed in Table 3.

**Table 3:** Glass-Transition Temperatures Determined by BDS after the Recrystallization of the Excess Amount of Drug from the NMS + 40 wt % KVA System at Various Temperatures and Corresponding Drug Concentrations of the NMS + KVA ASDs at 50 MPa

temperature of performed recrystallization [K]	368	378	388	398
*T*_g_ [K]	358 ± 2.1	357 ± 2.1	356 ± 1.7	354 ± 1.5
solubility limit (wt % of API in the system)	29 ± 1.4	31 ± 1.4	32 ± 1.1	36 ± 1.0

Finally, by comparing the obtained *T*_g_ values to the vertically shifted concentration dependence of the *T*_g_ values of the system obtained at ambient pressure (discussed earlier), we identified the solubility limits determined after the recrystallization of the excess amount of NMS from the supersaturated mixture [see panel (b) of Figure 9]. We listed the relevant values in Table 3. Based on the presented results, one can draw a conclusion that the solubility of the drug in the polymer matrix decreases with the increasing pressure. This is consistent with the recently published data.^37,38^

**Figure 10:**
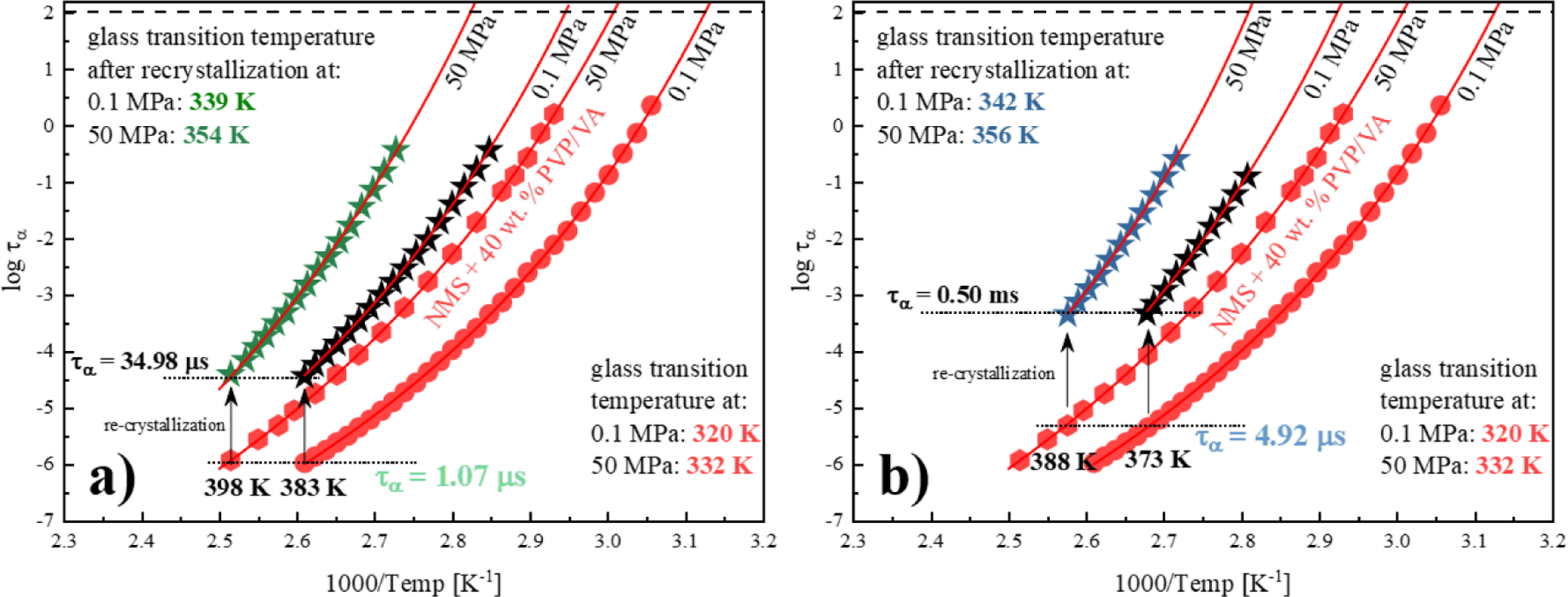
(a,b) τ_α_(*T*) registered for the fully amorphous NMS + 40 wt % KVA sample (red hexagons and circles for 50 and 0.1 MPa, respectively) as well as the sample after isothermal crystallization at: (i) 398 K and 50 MPa and (ii) 383 K and 0.1 MPa (green and black stars, respectively)—for panel (a); and (i) 388 K and 50 MPa and (ii) 373 K and 0.1 MPa (dark blue and black stars, respectively)—for panel (b). τ_α_(*T*) in the supercooled liquid has been described by the VFT equation (red solid lines).

By following the idea that the relaxation time is the key to sustain the desired level of solubility while increasing the pressure,^38^ we evaluated our results in this respect. By analyzing the obtained data, it is possible to observe instances where the recrystallization began for the same initial relaxation time: (i) 398 K and 50 MPa and 383 K and 0.1 MPa (τ_α_ = 1.07 μs); see panel (a) of Figure 10 and (ii) 388 K and 50 MPa and 373 K and 0.1 MPa (τ_α_ = 4.92 μs); see panel (b) of Figure 10.

**Figure 11:**
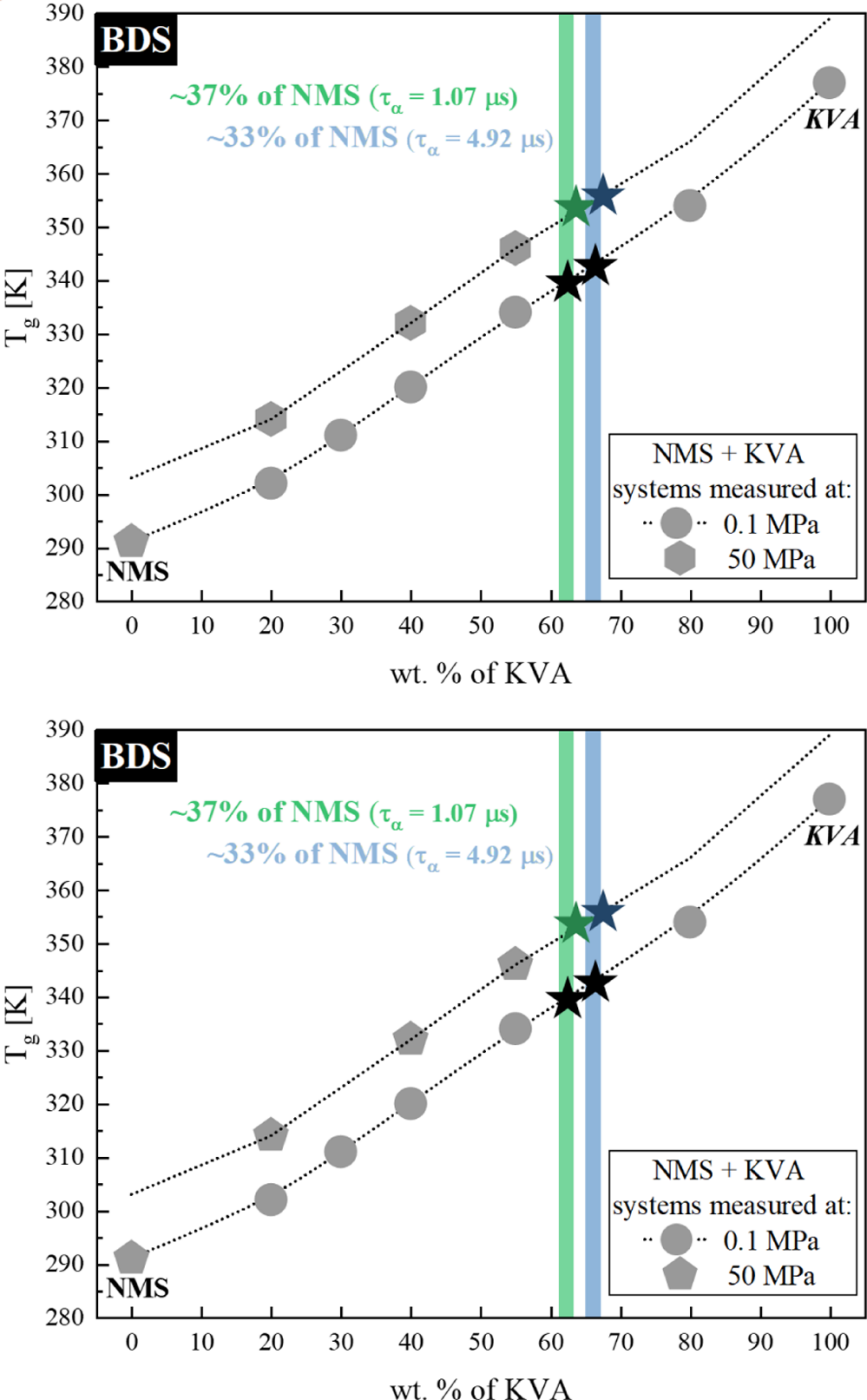
Experimentally determined glass-transition temperatures for the NMS + KVA mixtures. Hexagons and circles correspond to the experimentally determined *T*_g_ values at 50 and 0.1 MPa, respectively, while pentagons (NMS) represent the experimental data corresponding with the literature. Dotted line is the vertically transposed relationship to the *T*_g_ dependence obtained at ambient pressure. Stars correspond to the exact solubility limits determined during each measurement. Light green and light blue shadowed areas correspond to the concentrations obtained after recrystallization at isochronal, τ_α_ = 1.07 and 4.92 μs, conditions.

Figure 11 shows the solubility limit values obtained for the data above (recrystallization performed for the same initial relaxation time). Clearly, the presented result for the NMS + KVA ASD is yet another example, confirming that regardless of the chosen temperature or pressure conditions, there is a way of maintaining the desired level of solubility by sustaining the same initial relaxation time. This can contribute to a better understanding of the role of isochronal conditions in the solubility limit studies.

### Determination of the Solubility Limits of the NMS + KVA ASD at Elevated Pressure Conditions Using the FH Theory

**Figure 12:**
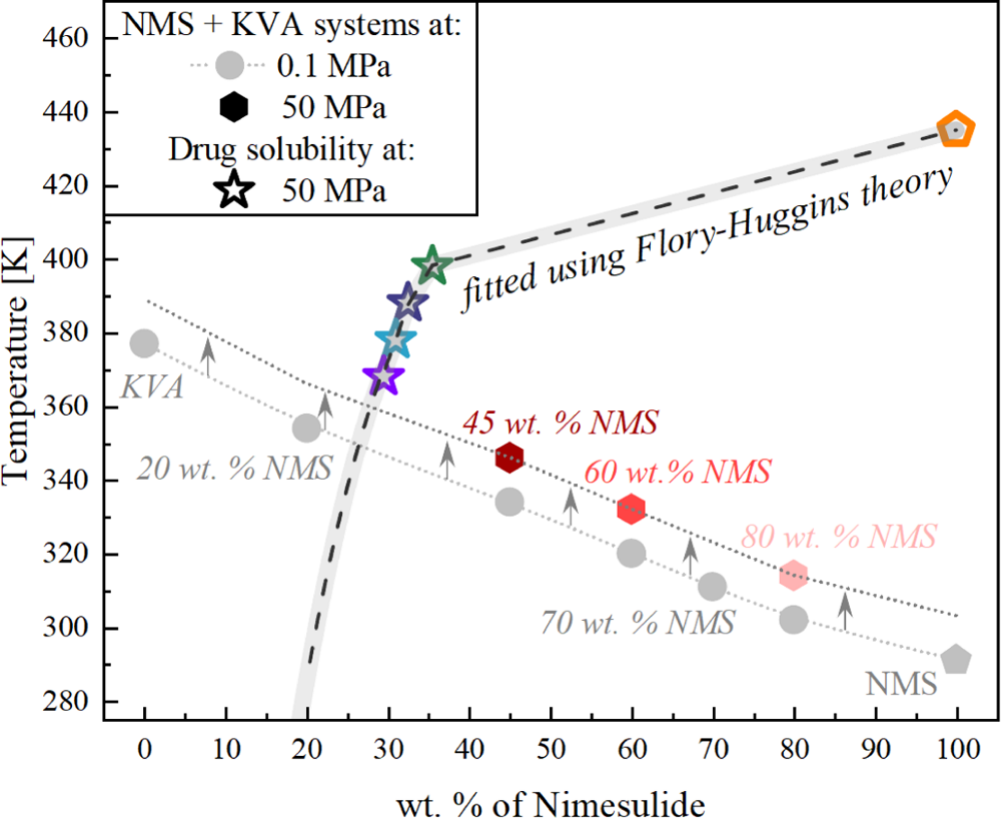
Glass-transition temperatures of NMS + KVA mixtures. Circles and hexagons correspond to the experimentally determined *T*_g_ at 0.1 and 50 MPa, respectively, while empty stars correspond to the solubility limit values determined at elevated pressures (50 MPa), and empty pentagons (neat NMS) represent data taken from the literature at elevated pressures.^65^ Dashed line represents the drug solubility in the polymer (the solid–liquid line) determined using the FH theory.

Finally, we applied the FH theory to the data generated under elevated pressure (50 MPa). The melting point of NMS under such a condition is 435 K,^65^ while the enthalpy of drug melting and density of the components were taken to be comparable to those obtained when no pressure was applied. Using the approach (I), the χ parameter was determined to be −1.3 ± 0.2, implying the miscibility of the drug and polymer; however, it was clear that the fit is not precise. Thus, the solubility of NMS in KVA at 298 K was found to be 3 wt %. Using the approach (II) and allowing the *A* and *B* parameters to fit best the experimental points, they now became 13.03 ± 0.3 and −5511 ± 113, respectively, and the solubility of API in the polymer was assessed to be 21.7 wt % (see Figure 12).

**Figure 13:**
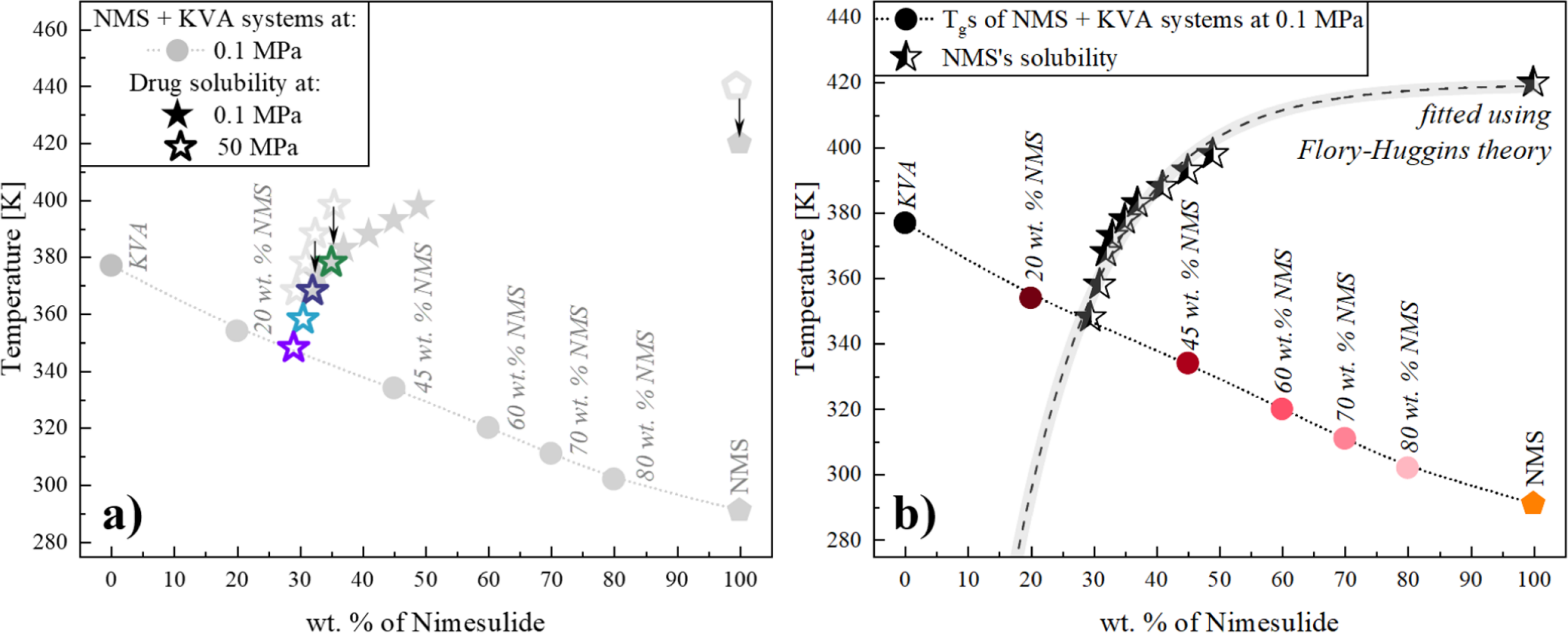
(a,b) Glass-transition temperatures of NMS + KVA mixtures. Circles correspond to the experimentally determined *T*_g_ at 0.1 MPa, while filled and empty stars correspond to the solubility limit values determined at ambient and elevated pressures (50 MPa), respectively, and filled and empty pentagons (NMS) represent data taken from the literature at ambient^47^ and elevated pressure,^65^ respectively. Panel (a) serves as a visual aid to illustrate the vertical transfer of the experimental data corresponding to the solubility limits obtained at elevated pressures. Panel (b) illustrates the data after the vertical transfer. Half-filled stars correspond to the combined data obtained at ambient and elevated pressures. Dashed line represents the drug’s solubility determined using the FH theory.

Next, we decided to shift vertically the experimental data corresponding to the solubility limits obtained at elevated pressures. As a reference point, we used the melting temperature of neat NMS at 50^65^ and 0.1 MPa.^47^ As can be seen in panel (a) of Figure 13, this procedure provides an interesting result (gray pentagons, empty and filled, respectively). The data overlap nearly seamlessly. As illustrated in panel (b) of Figure 13, the obtained set of data (generated by shifting the high-pressure points downward to the line formed by the experimental points at 0.1 MPa) was then fitted using the FH model. The “global” χ parameter for this data set was determined to be −1.41 ± 0.17, with the solubility of NMS at 298 K in the polymer equal to 3.7 wt %, slightly higher than the values obtained when the experimental points at 0.1 and 50 MPa were fitted separately. However, it needs to be highlighted that the quality of the fit as expressed by the *R*^2^ value was only 0.84. A better fit (*R*^2^ = 0.99) was determined using the approach (II), where the *A* and *B* parameters were calculated to be 10.85 ± 0.94 and −4602 ± 350, respectively, translating into NMS solubility of 22.6%, close to the value of solubility obtained for the high-pressure data alone. Thus, combining the measurements from atmospheric and high-pressure experiments can result in the widening of the data set and adding critical points at compositions not accessible when performing standard measurements and improving the predictions. Janssen et al. proposed that in relation to the pressure impact on *A* and *B*, the component related to the enthalpy of mixing (*B*) is not pressure-dependent, while the parameter *A*, correlated with the free volume of the mixture, should decrease as an increase in pressure reduces the free volume.^66^ This approach was tested here, and indeed, when the *B* parameter was kept constant at −2996, as determined for the atmospheric pressure data only (see above), the parameter *A* for the whole data set combined results obtained from both pressures, established at 5.825 ± 0.11, but it was not statistically different to that determined for the experimental points with no pressure effect. It appears that the 50 MPa pressure is not high enough to significantly change the parameter associated with a change in free volume, but as stated above, the data points generated by shifting the high-pressure points downward to the line formed by the experimental points at 0.1 MPa do improve the predictions.

Based on the presented results, one can draw a conclusion that despite the fact that the solubility at a given temperature changes with the applied pressure, the overall profile of the temperature dependence of the API solubility in the polymer matrix is not pressure-sensitive. Therefore, it would seem that by applying pressure, one can simply transpose this profile vertically. This approach allows to obtain the solubility values at elevated pressure conditions and transpose them to generate more reliable data for the data points at lower temperatures and at ambient pressure. This approach might be the long-awaited solution to the problem of experimental determination of the solubility limit of a drug in a polymer matrix at low temperatures. The possibility of determining data in an extended temperature range would further contribute to the further development and/or verification of theoretical models. However, this is only the first step toward the understanding of the role of elevated pressures in the solubility limit studies. Therefore, we strongly encourage further exploration of this phenomenon.

## Conclusions

To summarize, BDS investigations carried out on the NMS + KVAs systems provided results which were in excellent agreement with those obtained from calorimetric measurements in terms of the solubility limit determination. Furthermore, it was revealed that the solubility of the API in the polymer matrix decreased with increasing pressure [i.e. (i) 32 and 29 wt % at 368 K and 0.1 and 50 MPa, respectively or (ii) 49 and 36 wt % at 398 K and 0.1 and 50 MPa, respectively]. Experiments on the ASDs confirmed that regardless of the chosen sets of pressure or temperature conditions, the preservation of the same initial relaxation time is the key to maintaining the desired level of solubility, which will contribute to a better understanding of the role of the isochronal conditions in the solubility limit studies. Based on the presented data, one can conclude that by vertically transposing the results determined at elevated pressure, it is possible to obtain the solubility limit values corresponding to low temperatures. This may be the long-awaited solution to some of the limitations of experimental methods for determining drug solubility in a polymer matrix.

## References

[ref1] Newman A.; Knipp G.; Zografi G. Assessing the Performance of Amorphous Solid Dispersions. J. Pharm. Sci. 2012, 101, 1355–1377. 10.1002/jps.23031. 22213468

[ref2] Mahieu A.; Willart J.-F.; Dudognon E.; Danède F.; Descamps M. A New Protocol To Determine the Solubility of Drugs into Polymer Matrixes. Mol. Pharm. 2013, 10, 560–566. 10.1021/mp3002254. 23253068

[ref3] Lehmkemper K.; Kyeremateng S. O.; Degenhardt M.; Sadowski G. Influence of Low-Molecular-Weight Excipients on the Phase Behavior of PVPVA64 Amorphous Solid Dispersions. Pharm. Res. 2018, 35, 25 10.1007/s11095-017-2316-y. 29305717

[ref4] Rask M. B.; Knopp M. M.; Olesen N. E.; Holm R.; Rades T. Influence of PVP/VA Copolymer Composition on Drug–Polymer Solubility. Eur. J. Pharm. Sci. 2016, 85, 10–17. 10.1016/j.ejps.2016.01.026. 26826280

[ref5] Rask M. B.; Knopp M. M.; Olesen N. E.; Holm R.; Rades T. Comparison of Two DSC-Based Methods to Predict Drug-Polymer Solubility. Int. J. Pharm. 2018, 540, 98–105. 10.1016/j.ijpharm.2018.02.002. 29425764

[ref6] Li S.; Tian Y.; Jones D. S.; Andrews G. P. Optimising Drug Solubilisation in Amorphous Polymer Dispersions: Rational Selection of Hot-Melt Extrusion Processing Parameters. AAPS PharmSciTech 2016, 17, 200–213. 10.1208/s12249-015-0450-6. 26729536 PMC4766128

[ref7] Tamagawa R. E.; Martins W.; Derenzo S.; Bernardo A.; Rolemberg M. P.; Carvan P.; Giulietti M. Short-Cut Method To Predict the Solubility of Organic Molecules in Aqueous and Nonaqueous Solutions by Differential Scanning Calorimetry. Cryst. Growth Des. 2006, 6, 313–320. 10.1021/cg050128y.

[ref8] Nishi T.; Wang T. T. Melting Point Depression and Kinetic Effects of Cooling on Crystallization in Poly(Vinylidene Fluoride)-Poly(Methyl Methacrylate) Mixtures. Macromolecules 1975, 8, 909–915. 10.1021/ma60048a040.

[ref9] Sun Y.; Tao J.; Zhang G. G. Z.; Yu L. Solubilities of Crystalline Drugs in Polymers: An Improved Analytical Method and Comparison of Solubilities of Indomethacin and Nifedipine in PVP, PVP/VA, and PVAc. J. Pharm. Sci. 2010, 99, 4023–4031. 10.1002/jps.22251. 20607809

[ref10] Mohan R.; Lorenz H.; Myerson A. S. Solubility Measurement Using Differential Scanning Calorimetry. Ind. Eng. Chem. Res. 2002, 41, 4854–4862. 10.1021/ie0200353.

[ref11] Park K.; Evans J. M. B.; Myerson A. S. Determination of Solubility of Polymorphs Using Differential Scanning Calorimetry. Cryst. Growth Des. 2003, 3, 991–995. 10.1021/cg0340502.

[ref12] Qian F.; Huang J.; Hussain M. A. Drug–Polymer Solubility and Miscibility: Stability Consideration and Practical Challenges in Amorphous Solid Dispersion Development. J. Pharm. Sci. 2010, 99, 2941–2947. 10.1002/jps.22074. 20127825

[ref13] Tao J.; Sun Y.; Zhang G. G. Z.; Yu L. Solubility of Small-Molecule Crystals in Polymers: D-Mannitol in PVP, Indomethacin in PVP/VA, and Nifedipine in PVP/VA. Pharm. Res. 2009, 26, 855–864. 10.1007/s11095-008-9784-z. 19052850

[ref14] Gross J.; Sadowski G. Perturbed-Chain SAFT: An Equation of State Based on a Perturbation Theory for Chain Molecules. Ind. Eng. Chem. Res. 2001, 40, 1244–1260. 10.1021/ie0003887.

[ref15] Tihic A.; Kontogeorgis G. M.; von Solms N.; Michelsen M. L.; Constantinou L. A Predictive Group-Contribution Simplified PC-SAFT Equation of State: Application to Polymer Systems. Ind. Eng. Chem. Res. 2008, 47, 5092–5101. 10.1021/ie0710768.

[ref16] Gross J.; Sadowski G. Modeling Polymer Systems Using the Perturbed-Chain Statistical Associating Fluid Theory Equation of State. Ind. Eng. Chem. Res. 2002, 41, 1084–1093. 10.1021/ie010449g.

[ref17] Flory P. J. Thermodynamics of High Polymer Solutions. J. Chem. Phys. 1942, 10, 51–61. 10.1063/1.1723621.

[ref18] Zhao Y.; Inbar P.; Chokshi H. P.; Malick A. W.; Choi D. S. Prediction of the Thermal Phase Diagram of Amorphous Solid Dispersions by Flory-Huggins Theory. J. Pharm. Sci. 2011, 100, 3196–3207. 10.1002/jps.22541. 21416468

[ref19] Kozyra A.; Mugheirbi N. A.; Paluch K. J.; Garbacz G.; Tajber L. Phase Diagrams of Polymer-Dispersed Liquid Crystal Systems of Itraconazole/Component Immiscibility Induced by Molecular Anisotropy. Mol. Pharm. 2018, 15, 5192–5206. 10.1021/acs.molpharmaceut.8b00724. 30252481

[ref20] Potter C. B.; Davis M. T.; Albadarin A. B.; Walker G. M. Investigation of the Dependence of the Flory–Huggins Interaction Parameter on Temperature and Composition in a Drug–Polymer System. Mol. Pharm. 2018, 15, 5327–5335. 10.1021/acs.molpharmaceut.8b00797. 30259745

[ref21] Chakravarty P.; Lubach J. W.; Hau J.; Nagapudi K. A Rational Approach towards Development of Amorphous Solid Dispersions: Experimental and Computational Techniques. Int. J. Pharm. 2017, 519, 44–57. 10.1016/j.ijpharm.2017.01.003. 28063904

[ref22] Wlodarski K.; Tajber L.; Sawicki W. Physicochemical Properties of Direct Compression Tablets with Spray Dried and Ball Milled Solid Dispersions of Tadalafil in PVP-VA. Eur. J. Pharm. Biopharm. 2016, 109, 14–23. 10.1016/j.ejpb.2016.09.011. 27658987

[ref23] Crowley M. M.; Zhang F.; Koleng J. J.; McGinity J. W. Stability of Polyethylene Oxide in Matrix Tablets Prepared by Hot-Melt Extrusion. Biomaterials 2002, 23, 4241–4248. 10.1016/s0142-9612(02)00187-4. 12194527

[ref24] Crowley M. M.; Schroeder B.; Fredersdorf A.; Obara S.; Talarico M.; Kucera S.; McGinity J. W. Physicochemical Properties and Mechanism of Drug Release from Ethyl Cellulose Matrix Tablets Prepared by Direct Compression and Hot-Melt Extrusion. Int. J. Pharm. 2004, 269, 509–522. 10.1016/j.ijpharm.2003.09.037. 14706261

[ref25] Zhang F.; McGinity J. W. Properties of Hot-Melt Extruded Theophylline Tablets Containing Poly(Vinyl Acetate). Drug Dev. Ind. Pharm. 2000, 26, 931–942. 10.1081/ddc-100101320. 10914317

[ref26] Crowley M. M.; Zhang F.; Repka M. A.; Thumma S.; Upadhye S. B.; Kumar Battu S.; McGinity J. W.; Martin C. Pharmaceutical Applications of Hot-Melt Extrusion: Part I. Drug Dev. Ind. Pharm. 2007, 33, 909–926. 10.1080/03639040701498759. 17891577

[ref27] Repka M. A.; Battu S. K.; Upadhye S. B.; Thumma S.; Crowley M. M.; Zhang F.; Martin C.; McGinity J. W. Pharmaceutical Applications of Hot-Melt Extrusion: Part II. Drug Dev. Ind. Pharm. 2007, 33, 1043–1057. 10.1080/03639040701525627. 17963112

[ref28] Goff J.; Whelan T. The DYNISCO Extrusion Processors Handbook, 2nd ed.; DeLaney D., Ed.; DYNISCO, 2000.

[ref29] Thakral N. K.; Mohapatra S.; Stephenson G. A.; Suryanarayanan R. Compression-Induced Crystallization of Amorphous Indomethacin in Tablets: Characterization of Spatial Heterogeneity by Two-Dimensional X-Ray Diffractometry. Mol. Pharm. 2015, 12, 253–263. 10.1021/mp5005788. 25438193

[ref30] Berziņš K.; Suryanarayanan R. Compression-Induced Crystallization in Sucrose-Polyvinylpyrrolidone Amorphous Solid Dispersions. Cryst. Growth Des. 2018, 18, 839–848. 10.1021/acs.cgd.7b01305.

[ref31] Ayenew Z.; Paudel A.; Rombaut P.; Van Den Mooter G. Effect of Compression on Non-Isothermal Crystallization Behaviour of Amorphous Indomethacin. Pharm. Res. 2012, 29, 2489–2498. 10.1007/s11095-012-0778-5. 22638868

[ref32] Knapik-Kowalczuk J.; Wojnarowska Z.; Rams-Baron M.; Jurkiewicz K.; Cielecka-Piontek J.; Ngai K. L.; Paluch M. Atorvastatin as a Promising Crystallization Inhibitor of Amorphous Probucol: Dielectric Studies at Ambient and Elevated Pressure. Mol. Pharm. 2017, 14, 2670–2680. 10.1021/acs.molpharmaceut.7b00152. 28692796

[ref33] Kawakami K.; Ohba C. Crystallization of Probucol from Solution and the Glassy State. Int. J. Pharm. 2017, 517, 322–328. 10.1016/j.ijpharm.2016.12.027. 27979761

[ref34] Német Z.; Sztatisz J.; Demeter Á. Polymorph Transitions of Bicalutamide: A Remarkable Example of Mechanical Activation. J. Pharm. Sci. 2008, 97, 3222–3232. 10.1002/jps.21256. 18085711

[ref35] Szafraniec J.; Antosik A.; Knapik-Kowalczuk J.; Chmiel K.; Kurek M.; Gawlak K.; Paluch M.; Jachowicz R. Enhanced Dissolution of Solid Dispersions Containing Bicalutamide Subjected to Mechanical Stress. Int. J. Pharm. 2018, 542, 18 10.1016/j.ijpharm.2018.02.040. 29481948

[ref36] Szafraniec-Szczęsny J.; Antosik-Rogóż A.; Knapik-Kowalczuk J.; Kurek M.; Szefer E.; Gawlak K.; Chmiel K.; Peralta S.; Niwiński K.; Pielichowski K.; et al. Compression-Induced Phase Transitions of Bicalutamide. Pharmaceutics 2020, 12, 438 10.3390/pharmaceutics12050438. PMC7284452 32397432

[ref37] Chmiel K.; Knapik-Kowalczuk J.; Paluch M. How Does the High Pressure Affects the Solubility of the Drug within the Polymer Matrix in Solid Dispersion Systems. Eur. J. Pharm. Biopharm. 2019, 143, 8–17. 10.1016/j.ejpb.2019.08.003. 31398439

[ref38] Chmiel K.; Knapik-Kowalczuk J.; Paluch M. Isochronal Conditions-the Key to Maintain the given Solubility Limit, of a Small Molecule within the Polymer Matrix, at Elevated Pressure. Mol. Pharm. 2020, 17, 3730–3739. 10.1021/acs.molpharmaceut.0c00463. 32790413 PMC7539297

[ref39] Chmiel K.; Knapik-Kowalczuk J.; Jurkiewicz K.; Sawicki W.; Jachowicz R.; Paluch M. A New Method To Identify Physically Stable Concentration of Amorphous Solid Dispersions (I): Case of Flutamide + Kollidon VA64. Mol. Pharm. 2017, 14, 3370–3380. 10.1021/acs.molpharmaceut.7b00382. 28787567

[ref40] Chmiel K.; Knapik-Kowalczuk J.; Jachowicz R.; Paluch M. Broadband Dielectric Spectroscopy as an Experimental Alternative to Calorimetric Determination of the Solubility of Drugs into Polymer Matrix: Case of Flutamide and Various Polymeric Matrixes. Eur. J. Pharm. Biopharm. 2019, 136, 231–239. 10.1016/j.ejpb.2019.01.025. 30703545

[ref41] Knapik-Kowalczuk J.; Chmiel K.; Jurkiewicz K.; Wojnarowska Z.; Kurek M.; Jachowicz R.; Paluch M. Influence of Polymeric Additive on the Physical Stability and Viscoelastic Properties of Aripiprazole. Mol. Pharm. 2019, 16, 1742–1750. 10.1021/acs.molpharmaceut.9b00084. 30848603

[ref42] Pacult J.; Rams-Baron M.; Chmiel K.; Jurkiewicz K.; Antosik A.; Szafraniec J.; Kurek M.; Jachowicz R.; Paluch M. How Can We Improve the Physical Stability of Co-Amorphous System Containing Flutamide and Bicalutamide? The Case of Ternary Amorphous Solid Dispersions. Eur. J. Pharm. Sci. 2019, 136, 104947 10.1016/j.ejps.2019.06.001. 31170526

[ref43] Knapik-Kowalczuk J.; Chmiel K.; Pacułt J.; Bialek K.; Tajber L.; Paluch M. Enhancement of the Physical Stability of Amorphous Sildenafil in a Binary Mixture, with Either a Plasticizing or Antiplasticizing Compound. Pharmaceutics 2020, 12, 460 10.3390/pharmaceutics12050460. PMC7284710 32443637

[ref44] Rams-Baron M.; Pacułt J.; Jędrzejowska A.; Knapik-Kowalczuk J.; Paluch M. Changes in Physical Stability of Supercooled Etoricoxib after Compression. Mol. Pharm. 2018, 15, 3969–3978. 10.1021/acs.molpharmaceut.8b00428. 30052449

[ref45] Knapik-Kowalczuk J.; Chmiel K.; Paluch M. Crystallization of Amorphous Pharmaceuticals at Ambient and Elevated Pressure Conditions. Crystallization as Studied by Broadband Dielectric Spectroscopy; Springer: Cham, 2020; pp 55–87.

[ref46] Jensen M. H.; Alba-Simionesco C.; Niss K.; Hecksher T. A Systematic Study of the Isothermal Crystallization of the Mono-Alcohol n-Butanol Monitored by Dielectric Spectroscopy. J. Chem. Phys. 2015, 143, 134501 10.1063/1.4931807. 26450317

[ref47] Knapik J.; Wojnarowska Z.; Grzybowska K.; Tajber L.; Mesallati H.; Paluch K. J.; Paluch M. Molecular Dynamics and Physical Stability of Amorphous Nimesulide Drug and Its Binary Drug-Polymer Systems. Mol. Pharm. 2016, 13, 1937–1946. 10.1021/acs.molpharmaceut.6b00115. 27149568

[ref48] Baird J. A.; Taylor L. S. Evaluation of Amorphous Solid Dispersion Properties Using Thermal Analysis Techniques. Advanced Drug Delivery Reviews; Elsevier, April 1, 2012; pp 396–421. 10.1016/j.addr.2011.07.009 21843564

[ref49] Romanini M.; Lorente M.; Schammé B. S.; Delbreilh L.; Dupray V. V.; Coquerel G. G.; Lluís Tamarit J.; Macovez R. Enhancement of the Physical and Chemical Stability of Amorphous Drug–Polymer Mixtures via Cryogenic Comilling. Macromolecules 2018, 51, 9382 10.1021/acs.macromol.8b01271.

[ref50] Knapik J.; Wojnarowska Z.; Grzybowska K.; Jurkiewicz K.; Tajber L.; Paluch M. Molecular Dynamics and Physical Stability of Coamorphous Ezetimib and Indapamide Mixtures. Mol. Pharm. 2015, 12, 3610–3619. 10.1021/acs.molpharmaceut.5b00334. 26301858

[ref51] Van den Mooter G.; Wuyts M.; Blaton N.; Busson R.; Grobet P.; Augustijns P.; Kinget R. Physical Stabilisation of Amorphous Ketoconazole in Solid Dispersions with Polyvinylpyrrolidone K25. Eur. J. Pharm. Sci. 2001, 12, 261–269. 10.1016/s0928-0987(00)00173-1. 11113645

[ref52] Baghel S.; Cathcart H.; O’Reilly N. J. Polymeric Amorphous Solid Dispersions: A Review of Amorphization, Crystallization, Stabilization, Solid-State Characterization, and Aqueous Solubilization of Biopharmaceutical Classification System Class II Drugs. J. Pharm. Sci. 2016, 105, 2527–2544. 10.1016/j.xphs.2015.10.008. 26886314

[ref53] Rams-Baron M.; Wojnarowska Z.; Grzybowska K.; Dulski M.; Knapik J.; Jurkiewicz K.; Smolka W.; Sawicki W.; Ratuszna A.; Paluch M. Toward a Better Understanding of the Physical Stability of Amorphous Anti-Inflammatory Agents: The Roles of Molecular Mobility and Molecular Interaction Patterns. Mol. Pharm. 2015, 12, 3628–3638. 10.1021/acs.molpharmaceut.5b00351. 26323061

[ref54] Knapik J.; Wojnarowska Z.; Grzybowska K.; Jurkiewicz K.; Stankiewicz A.; Paluch M. Stabilization of the Amorphous Ezetimibe Drug by Confining Its Dimension. Mol. Pharm. 2016, 13, 1308–1316. 10.1021/acs.molpharmaceut.5b00903. 26981876

[ref55] Kremer F.; Schönhals A.; Kremer F. Broadband Dielectric Spectroscopy; Springer: Berlin, Heidelberg, 2003.

[ref56] Vogel H. Temperaturabhangigkeitgesetz Der Viskosität von Flüssigkeiten. J. Phys. Z. 1921, 22, 645–646.

[ref57] Fulcher G. S. Analysis of Recent Measurements of the Viscosity of Glasses. J. Am. Ceram. Soc. 1925, 8, 339–355. 10.1111/j.1151-2916.1925.tb16731.x.

[ref58] Tammann G.; Hesse W. Die Abhängigkeit der Viscosität von der Temperatur bie unterkühlten Flüssigkeiten. Z. Anorg. Allg. Chem. 1926, 156, 245–257. 10.1002/zaac.19261560121.

[ref59] Phan A. D.; Thu Thuy T. T.; Kim An N. T.; Knapik-Kowalczuk J.; Paluch M.; Wakabayashi K. Molecular Relaxations in Supercooled Liquid and Glassy States of Amorphous Gambogic Acid: Dielectric Spectroscopy, Calorimetry, and Theoretical Approach. AIP Adv. 2020, 10, 025128 10.1063/1.5139101.

[ref60] Marsac P. J.; Li T.; Taylor L. S. Estimation of Drug-Polymer Miscibility and Solubility in Amorphous Solid Dispersions Using Experimentally Determined Interaction Parameters. Pharm. Res. 2009, 26, 139–151. 10.1007/s11095-008-9721-1. 18779927

[ref61] Knopp M. M.; Tajber L.; Tian Y.; Olesen N. E.; Jones D. S.; Kozyra A.; Löbmann K.; Paluch K.; Brennan C. M.; Holm R.; et al. Comparative Study of Different Methods for the Prediction of Drug–Polymer Solubility. Mol. Pharm. 2015, 12, 3408–3419. 10.1021/acs.molpharmaceut.5b00423. 26214347

[ref62] Rubinstein M.; Colby R. H. Polymer Physics; Oxford University Press: Oxford, UK, 2003.

[ref63] Hemley R. J. Effects of High Pressure on Molecules. Annu. Rev. Phys. Chem. 2000, 51, 763–800. 10.1146/annurev.physchem.51.1.763. 11031299

[ref64] Paluch M.; Rzoska S. J.; Habdas P.; Ziolo J. On the Isothermal Pressure Behaviour of the Relaxation Times for Supercooled Glass-Forming Liquids. J. Phys.: Condens. Matter 1998, 10, 4131–4138. 10.1088/0953-8984/10/19/001.

[ref65] Barrio M.; Huguet J.; Robert B.; Rietveld I. B.; Céolin R.; Tamarit J. L. Pressure-Temperature Phase Diagram of the Dimorphism of the Anti-Inflammatory Drug Nimesulide. Int. J. Pharm. 2017, 525, 54–59. 10.1016/j.ijpharm.2017.04.016. 28411142

[ref66] Janssen S.; Schwahn D.; Mortensen K.; Springer T. Pressure Dependence of the Flory-Huggins Interaction Parameter in Polymer Blends: A SANS Study and a Comparison to the Flory-Orwoll-Vrij Equation of State. Macromolecules 1993, 26, 5587–5591. 10.1021/ma00073a009.

